# Human Striatal Response to Reward Anticipation Linked to Hippocampal Glutamate Levels

**DOI:** 10.1093/ijnp/pyy011

**Published:** 2018-02-10

**Authors:** Matthijs G Bossong, Robin Wilson, Elizabeth Appiah-Kusi, Philip McGuire, Sagnik Bhattacharyya

**Affiliations:** 1 Department of Psychosis Studies, Institute of Psychiatry, Psychology and Neuroscience, King’s College London, United Kingdom; 2 Department of Psychiatry, Brain Center Rudolf Magnus, University Medical Center Utrecht, The Netherlands

**Keywords:** reward, striatum, hippocampus, glutamate, neuroimaging

## Abstract

**Background:**

Dysfunctional reward processing is associated with a number of psychiatric disorders, such as addiction and schizophrenia. It is thought that reward is regulated mainly by dopamine transmission in the ventral striatum. Contemporary animal models suggest that striatal dopamine concentrations and associated behaviors are related to glutamatergic functioning in the ventral hippocampus. However, in humans the association between reward-related ventral striatal response and hippocampal glutamate levels is unclear.

**Methods:**

Nineteen healthy participants were studied using proton magnetic resonance spectroscopy to measure hippocampal glutamate levels, and functional magnetic resonance imaging to assess striatal activation and functional connectivity during performance of a monetary incentive delay task.

**Results:**

We found that ventral striatal activation related to reward processing was correlated with hippocampal glutamate levels. In addition, context-dependent functional coupling was demonstrated between the ventral striatum and both the lingual gyrus and hippocampus during reward anticipation. Elevated hippocampal glutamate levels were inversely related to context-dependent functional connectivity between the ventral striatum and the anterior hippocampus while anticipating reward.

**Conclusions:**

These findings indicate that human striatal responses to reward are influenced by hippocampal glutamate levels. This may be relevant for psychiatric disorders associated with abnormal reward processing such as addiction and schizophrenia.

**Significance Statement**

Rewards are a key determinant of whether we eat, drink, or mate. Dysfunctional reward processing is associated with a number of psychiatric disorders, such as schizophrenia and addiction. It is thought that reward processing is regulated mainly by dopamine transmission in the ventral striatum. Animal models suggest that striatal dopamine concentrations and associated behaviors are related to glutamatergic functioning in the ventral hippocampus. Here, we investigated the relationship between hippocampal glutamate levels and the ventral striatal response to reward anticipation in humans. We found that higher hippocampal glutamate levels are correlated with reward-related ventral striatal activity, but inversely correlated with functional connectivity between anterior hippocampus and ventral striatum during reward anticipation. This suggests that human striatal responses to reward anticipation are influenced by hippocampal glutamate levels. This may be relevant for psychiatric disorders associated with abnormal reward processing such as addiction and schizophrenia.

## Introduction

Rewards are a key determinant of whether we eat, drink, or mate (Schultz, 2015). Reward in this context refers to the attractive and motivational property of a stimulus that induces goal-directed behavior (Berridge and Kringelbach, 2015; Schultz, 2015). Dysfunctional reward processing is associated with a number of psychiatric disorders, such as schizophrenia and addiction (Berridge, 2012; Berridge and Kringelbach, 2015; Radua et al., 2015; Luijten et al., 2017), wherein it is thought that inappropriate attribution of incentive salience to otherwise relatively neutral environmental cues result in the formation of psychotic symptoms (Heinz, 2002; Kapur, 2003) or the development of addictive behavior (Flagel et al., 2009; Berridge, 2012).

Reward processing is primarily regulated by the mesolimbic dopamine system, which, in animals, originates in the ventral tegmental area (VTA) and projects to the nucleus accumbens (Berridge, 2012; Berridge and Kringelbach, 2015; Schultz, 2015). Numerous animal studies have shown increased midbrain dopaminergic activity and elevated dopamine levels in the nucleus accumbens in relation to reward anticipation (Schultz et al.,1997; Roitman et al., 2004; Schultz, 2015). In human neuroimaging research, reward processing has frequently been investigated during performance of a monetary incentive delay (MID) task that involves reward anticipation and receipt (Knutson et al., 2001; Bjork et al., 2004; van Hell et al., 2012; Jansma et al., 2013; Radua et al., 2015; Luijten et al., 2017). Results of studies using this task in healthy individuals have identified the ventral striatum, a brain structure predominantly comprising the nucleus accumbens, being critically involved in reward processing (Knutson et al, 2001; Bjork et al., 2004; Knutson and Cooper, 2005). Furthermore, increased ventral striatal activity during reward processing has been shown to be related to dopamine release in this brain region (Knutson and Gibbs, 2007; Schott et al., 2008).

Preclinical models suggest that striatal dopamine levels and associated behaviors are related to the functioning of the ventral hippocampus (Lodge and Grace, 2011; Grace, 2016). For example, stimulation of the ventral hippocampus produces robust and sustained increases in extracellular dopamine concentrations in the nucleus accumbens (Blaha et al., 1997; Legault and Wise, 1999). In addition, experimental activation of glutamatergic N-methyl-D-aspartate receptors in the ventral hippocampus dramatically increases dopamine neuron activity in the VTA in a dose-dependent manner (Floresco et al., 2001, 2003; Lodge and Grace, 2006). This activation of the hippocampal glutamate system is directly correlated with both dopamine release in the nucleus accumbens (Floresco et al., 2003) and the behavioral response to amphetamine (White et al., 2006; Lodge and Grace, 2008). This cascade of activated glutamatergic pyramidal neurons driving increased striatal dopamine levels is thought to be controlled by parvalbumin-expressing GABAergic interneurons in the ventral hippocampus, with reduced inhibitory control of glutamatergic pyramidal neurons leading to higher striatal dopamine concentrations (Lodge and Grace, 2011; Grace, 2016).

Only a limited number of human neuroimaging studies have examined interactions between hippocampal and striatal function in healthy participants. Stone and colleagues (2010) examined hippocampal glutamate levels measured with Proton Magnetic Resonance Spectroscopy (^1^H-MRS) and striatal dopamine synthesis capacity measured with Positron Emission Tomography in 12 healthy subjects but did not find a significant correlation (although they did report a correlation in individuals at high risk for psychosis). A study by Allen et al. (2012) in 14 healthy participants found that greater activation in the hippocampus during performance of a verbal memory task was associated with diminished striatal dopamine synthesis capacity. Finally, Roiser and colleagues (2013) described a positive correlation between hippocampal responses to irrelevant stimulus features and striatal dopamine synthesis capacity in 18 healthy subjects. However, the relationship between hippocampal glutamate levels and ventral striatal activity in the context of reward processing is unclear.

The aim of the present study was to examine the relationship between hippocampal glutamate levels and the ventral striatal response to reward anticipation in humans. Nineteen healthy participants were studied using ^1^H-MRS to measure hippocampal glutamate levels, and functional MRI to assess striatal activation and functional connectivity during performance of the MID task. Following contemporary animal models (Lodge and Grace, 2011; Grace, 2016), we hypothesized that increased hippocampal glutamate levels would be associated with higher activation in the ventral striatum during the anticipation of monetary reward. Because these preclinical models describe reduced inhibitory control of glutamatergic pyramidal neurons leading to higher striatal dopamine concentrations, a further prediction was that increased hippocampal glutamate levels would also be related to reduced reward-related functional coupling between the hippocampus and ventral striatum.

## Methods

### Participants

Nineteen healthy volunteers participated in the study. They were recruited through advertisements on websites. The mean age of the subjects was 25.8±5.6 years (range 20–38), and 10 were male and 9 were female. Their self-reported ethnicity was white British (n=7), black (n=2), Asian (n=6), and mixed (n=4). Mean total years of education was 15.1±3.0. All participants were right-handed and had no history of neurological or psychiatric disorder, or drug or alcohol dependence. The study had National Health Service UK Research Ethics Committee approval, and all participants gave informed consent.

### Reward Paradigm

**Figure 1.**
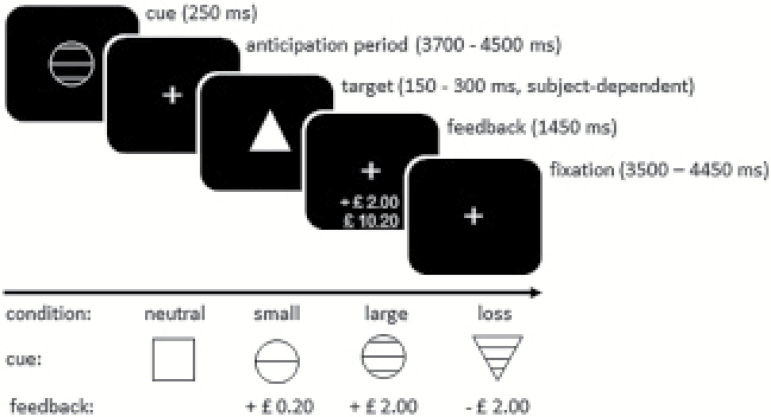
Reward paradigm. Each trial started with the presentation of a cue signalling a neutral, reward (small or large) or loss avoidance trial. After the cue, a target was presented to which subjects had to respond as fast as possible by pressing a button. At the end of each trial, visual feedback on performance was provided. The time between cue and target (anticipation phase) was varied between trials (3700–4500 ms). The inter-trial interval was 10 s for all trials.

To activate reward circuitry, an adapted version of the MID task as developed by Knutson and colleagues was used (Knutson et al., 2001). In this task, subjects are required to press a button as fast as possible on seeing a target stimulus. Depending on the cue that precedes the target stimulus, subjects can either win or avoid the loss of a certain amount of money. After each trial, subjects are given visual feedback about the amount won or lost in that trial, as well as the total amount won (Figure 1). The MID task consisted of 4 conditions: neutral, small reward (20 pence), large reward (2 pounds), and loss avoidance (2 pounds). There were 12 trials for each condition. The neutral condition was used as the control condition. Total task duration was 16 min, which was scanned in 2 consecutive 8-min runs. Monetary reward earned by subjects was related to actual task performance, starting with 10 pounds.

The reward cue was presented for 250 ms, while the feedback was presented for 1450 ms. A correct response was defined as a response before the target disappeared, but not earlier than 100 ms after its appearance. All other responses were considered incorrect. Initial target presentation time was 250 ms, but this was individually adapted (±10 ms, with a minimum of 150 ms and a maximum of 300 ms) to ensure approximately 66% accuracy for each subject. Intervals between the cue and target (the anticipation phase) varied between 3700 and 4500 ms. The inter-trial interval was 10 s for all trials (see Figure 1).

### Image Acquisition

All subjects underwent structural MRI, functional MRI, and ^1^H-MRS scanning in one session. Images were acquired on a General Electric 3.0 Tesla HDx MR system.

#### Structural MRI

Structural images were acquired using a whole-brain 3-dimensional sagittal T1-weighted scan, with parameters based on the Alzheimer’s Disease Neuroimaging Initiative (TE=2.85 ms; TR=6.98 ms; inversion time=400 ms; flip angle=11º; voxel size 1.0x1.0x1.2 mm; for full details, see http://adni.loni.usc.edu/methods/mri-analysis/mri-acquisition/).

#### Functional MRI

A total 480 T2*-weighted images were acquired in 2 runs of 8 min each with TE=30 ms, TR=2.0 s, and flip angle=75° in 39 axial planes (3 mm thick with an inter-slice gap of 3.3 mm), with an in-plane voxel size of 3.75 x 3.75 mm.

#### ^1^H-MRS

^1^H-MRS spectra (PRESS—Point RESolved Spectroscopy; TE=30 ms; TR=3000 ms; 96 averages) were acquired in the left hippocampus, as previously described by Stone et al. (2009). We employed the standard GE probe (proton brain examination) sequence, which uses a standardized chemically selective suppression water suppression routine. For each metabolite spectrum, unsuppressed water reference spectra (16 averages) were also acquired as part of the standard acquisition. Shimming and water suppression were optimized, with auto-prescan performed twice before each scan. Using standardized protocols, the hippocampal region of interest (ROI) (20 x 20 x 15 mm; right-left, anterior-posterior, superior-inferior) was prescribed from the structural T1 scan.

### Data Analysis

#### Task Performance

Performance accuracy (mean percentage of correct responses) and reaction time were examined using a repeated-measures ANOVA with task condition (4 levels: neutral, small reward, large reward, and loss avoidance) as within-subject factor. Posthoc analysis was performed with paired sample *t* tests.

#### Reward Processing

Functional MRI data were preprocessed and analyzed using SPM8 (Wellcome Trust Centre for Neuroimaging). Preprocessing included realignment of functional images, co-registration with the anatomical scan, spatial normalization into standard MNI space, and smoothing with a Gaussian filter (FWHM=8 mm).

For each subject, regression coefficients for each voxel were obtained from a general linear model regression analysis with factors time-locked to task events, convolved with a canonical hemodynamic response function. The design included a total of 13 regressors. Four regressors modelled anticipation activity for each of the 4 conditions. Eight regressors modelled the feedback activity, 1 for correct and 1 for incorrect responses for each of the 4 conditions. Finally, 1 regressor modelled response activity for all the 4 conditions. Group activity maps for reward anticipation were created, contrasting activation during rewarding task conditions (both small and large reward) to that during control conditions (neutral). We focus on reward anticipation because this is shown to depend on striatal dopamine function (Knutson and Gibbs, 2007; Schott et al., 2008). Brain activation was examined in the bilateral striatum using a mask consisting of caudate, pallidum, and putamen, as defined in the AAL atlas provided in SPM8. Results were FWE-corrected for the number of voxels in the bilateral striatum (*P<*.05).

#### Functional Connectivity

**Figure 2.**
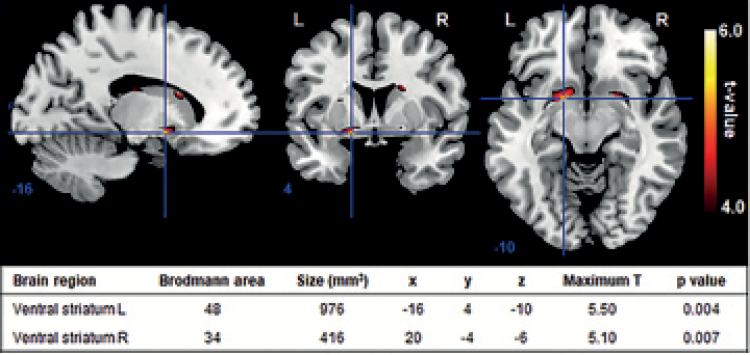
Group activity map for anticipation vs control shows significant activation in bilateral ventral striatum (n=19; *P<*.05, FWE-corrected for number of voxels in bilateral striatum). Numbers below slices indicate Montreal Neurological Institute xyz coordinates. L, left; R, right.

Functional connectivity analyses were performed using psychophysiological interaction (PPI) analysis approach (Friston et al., 1997) to examine the functional coupling during rewarding task conditions (both small and large reward) vs control conditions (neutral) (i.e., psychological factor). The cluster in the left ventral striatum that was significantly activated during reward anticipation (Figure 2) was used as the seed region. The left ventral striatum was selected as seed region, because ^1^H-MRS spectra were acquired in the left hippocampus, and the anatomical projection from primate hippocampus to ventral striatum is predominantly ipsilateral (Friedman et al., 2002). For each subject, the first eigenvariate of the blood oxygen level-dependent signal within the seed region was determined, and the interaction between activity within the seed region and the psychological factor (i.e. PPI regressor) was calculated. Individual contrast images were then created showing voxel-wise correlations with ventral striatal activity during reward processing. Subsequently, these individual maps of the PPI analyses were entered into a group analysis to examine functional connectivity with the left ventral striatum during reward anticipation. Whole-brain voxel-wise analyses were performed, and results were FWE corrected at cluster level (*P<*.05).

#### ^1^H-MRS Quantification

All spectra were analyzed with LCModel version 6.3-0A (Provencher, 1993) using a standard basis set of 16 metabolites (L-alanine, aspartate, creatine, phosphocreatine, GABA, glucose, glutamine, glutamate, glycerophosphocholine, glycine, myo-inositol, L-lactate, N-acetylaspartate, N-acetylaspartylglutamate, phosphocholine, and taurine), acquired with the same field strength (3 Tesla), localization sequence (PRESS), and echo time (30 ms). Model metabolites and concentrations used in the basis set are fully detailed in the LCModel manual (http://s-provencher-.com/pages/lcmmanual.shtml). Poorly fitted metabolite peaks (Cramer-Rao minimum variance bounds of >20% as reported by LCModel) were excluded from further analysis. Values of the combined water-scaled measure of glutamate and glutamine (Glx) were corrected for CSF content of the ROI using the formula Mcorr=M*(WM + GM + 1.55 CSF)/(WM + GM), where M is the uncorrected metabolite value, and WM, GM, and CSF are the white matter, grey matter, and CSF fractions of the ROI, respectively (Egerton et al., 2014). These fractions were determined for each subject from the structural T1 scans, which were used to localize the spectroscopy ROIs and subsequently segmented into GM, WM, and CSF using SPM8. The composite Glx peak has been widely used as a marker of glutamatergic function, because it likely predominantly reflects glutamate levels, which are typically 5 to 6 times higher than those of glutamine (Kaiser et al., 2005).

#### Correlations

For every subject, ventral striatal activity during reward anticipation was determined by extracting regression coefficients (b values) from the significantly activated cluster in the left ventral striatum (Figure 2). Subsequently, for every subject, functional connectivity between hippocampus and ventral striatum during reward anticipation was assessed by extracting connectivity coefficients resulting from the PPI analysis from the left anterior hippocampus, as defined in the AAL atlas. We focus on the anterior hippocampus, because in humans this brain area is functionally equivalent to the ventral hippocampus described in relevant preclinical models (Grace, 2012, 2017). Extraction of data was performed using the Marsbar SPM tool (Brett et al., 2002). Hypotheses on the correlations between hippocampal glutamate levels and (1) activity in the left ventral striatum, and (2) connectivity between left ventral striatum and left anterior hippocampus were tested using Pearson’s correlation (1-sided).

## Results

### Task Performance

Task condition had a significant effect on accuracy and reaction time (F(3,54)=13.18, *P<*.001 and F(3,54)=16.67, *P<*.001, respectively). Accuracy on the neutral task condition (54.4±10.4%) was significantly lower than that on loss avoidance (69.8±8.6%), small reward (63.4±8.9%), and large reward conditions (70.8±11.8%) (all *P<*.005). Reaction times were significantly higher for neutral trials (255±28 ms) compared with loss avoidance (232±26 ms), small reward (239±27 ms), and large reward trials (231±33 ms) (all *P<*.001). Accuracy and reaction times for small reward conditions were significantly different from those for loss avoidance and large reward conditions (all *P<*.05). There were no significant differences in task performance between loss avoidance and large reward trials.

### Ventral Striatal Activity During Reward Anticipation

The group map for anticipation vs control showed significant activity in the bilateral ventral striatum (*P<*.05, FWE-corrected for number of voxels in bilateral striatum; Figure 2).

#### PPI With Left Ventral Striatum as Seed Region

**Figure 3.**
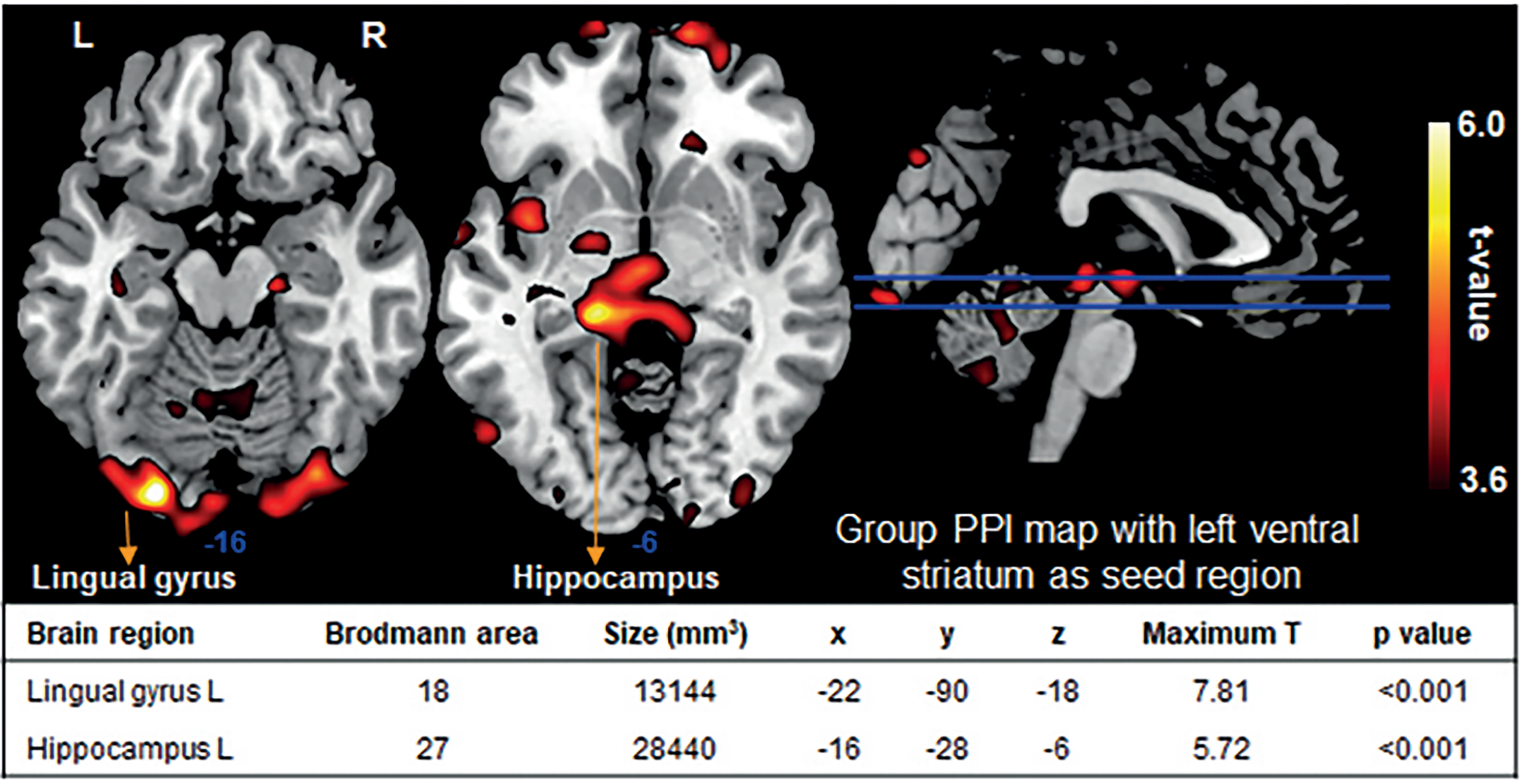
Group psychophysiological interaction (PPI) map for anticipation vs control with left ventral striatum as seed region (n=19; *P<*.001 uncorrected). Activation in left ventral striatum was significantly correlated with that in left lingual gyrus and left hippocampus (*P<*.001, FWE-corrected at cluster level). X, y, and z are Montreal Neurological Institute coordinates and represent the highest *t* value in a cluster. Numbers below slices indicate Montreal Neurological Institute z coordinates. L, left; R, right.

Context-dependent functional activity in the left ventral striatum (reward anticipation>control) was significantly correlated with that in the left lingual gyrus and left hippocampus (*P<*.001, FWE-corrected at cluster level; Figure 3), suggesting context-dependent functional coupling between the left ventral striatum and these regions.

### Hippocampal Glutamate Measures

In this group of healthy controls, the mean combined measure of glutamate and glutamine (Glx) in the left hippocampus was 9.95±1.96. The hippocampal spectroscopic voxel consisted of 4±1% CSF, 66±5% GM, and 30±6% WM. Spectral quality as reported by LCModel were (mean±SD): signal-to-noise ratio: 14±2; line width: 8.1±1.4.

### Correlations

**Figure 4.**
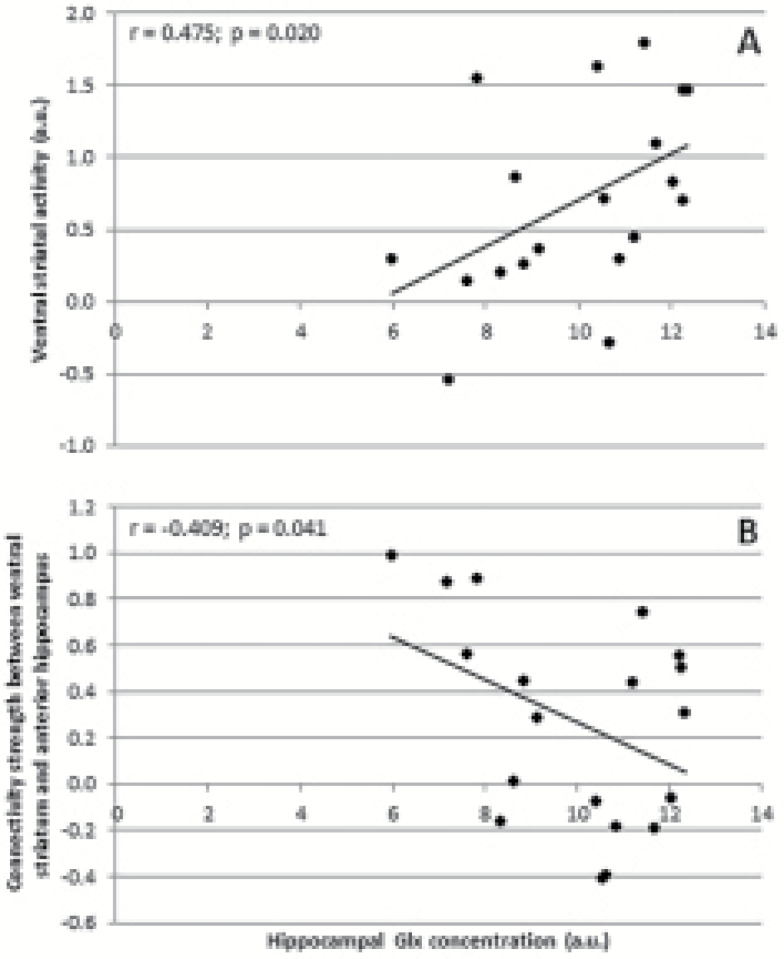
Correlation between left hippocampal Glx concentrations and (A) activity in left ventral striatum, and (B) connectivity between left ventral striatum and left anterior hippocampus, both for reward anticipation vs control. a.u.,arbitrary units.

Left hippocampal Glx concentrations showed a significant positive correlation with functional activity in the left ventral striatum during reward anticipation (r=0.475, *P=*.020; Figure 4A), and a significant negative correlation with context-dependent functional connectivity between the left ventral striatum and the left anterior hippocampus during reward anticipation (r=–0.409, *P=*.041; Figure 4B).

## Discussion

This is the first human neuroimaging study examining the relationship between hippocampal glutamate concentrations and ventral striatal reward processing. Our main findings were that hippocampal glutamate levels were correlated with reward anticipation-related ventral striatal activity, but inversely correlated with context-dependent functional connectivity between the anterior hippocampus and the ventral striatum during the anticipation of monetary reward. These findings suggest that in the context of anticipating a reward, the higher the level of glutamate in the hippocampus, the greater the ventral striatal response to rewarding stimuli. Concomitantly, the higher the level of glutamate in the hippocampus, the lower the functional coupling between hippocampus and ventral striatum while anticipating monetary reward. This suggests an inverse relationship between hippocampal glutamate levels and its control over ventral striatal function in the context of processing rewarding stimuli, possibly indicating that increased hippocampal glutamate levels are associated with reduced hippocampal control of striatal response to reward. Overall, the findings from the present study provide the first evidence that human striatal responses to reward anticipation are influenced by hippocampal glutamate levels. This may be highly relevant for psychiatric disorders associated with abnormal reward processing such as addiction and schizophrenia.

Our results are consistent with accumulating evidence from animal models that suggests that striatal dopamine levels and associated behaviors are related to functioning of the ventral hippocampus (Lodge and Grace, 2011; Grace, 2016). For example, activation of glutamatergic N-methyl-D-aspartate receptors in the ventral hippocampus elevates dopamine neuron activity in the VTA in a dose-dependent manner (Floresco et al., 2001, 2003; Lodge and Grace, 2006), which is correlated with both altered dopamine efflux in the nucleus accumbens (Floresco et al., 2003) and an increased behavioral response to amphetamine (White et al., 2006; Lodge and Grace, 2008). One possible explanation for the inverse correlation between hippocampal glutamate levels and reward-related functional connectivity between the anterior hippocampus and the ventral striatum that we observed in the present study is that this may reflect the relationship between activated glutamatergic pyramidal neurons leading to increased striatal dopamine levels and its control by inhibitory parvalbumin-expressing GABAergic interneurons in the anterior hippocampus. This is supported by preclinical models, which showed that reduced inhibitory control in the ventral hippocampus can lead to increased activation of glutamatergic pyramidal neurons, increased dopamine neuron activity in the VTA, and greater dopamine release in the nucleus accumbens (Lodge and Grace, 2011; Grace, 2016).

Three human neuroimaging studies have previously examined interactions between hippocampal and striatal function in healthy participants. Allen et al. (2012) demonstrated that higher activity in the left hippocampus during performance of a verbal memory task was associated with reduced ventral striatal dopamine synthesis capacity. Roiser and colleagues (2013) showed a significant positive correlation between right hippocampal activity in response to irrelevant stimulus features and striatal dopamine synthesis capacity, although this was present only in the dorsal striatum. The only previous study that has assessed the relationship between glutamate levels in the hippocampus and striatal dopamine function was conducted by Stone and colleagues (2010). They did not find a significant correlation between hippocampal glutamate levels as measured with ^1^H-MRS and striatal dopamine synthesis capacity assessed with Positron Emission Tomography, although they did find a correlation in subjects at clinical high risk for psychosis. None of these previous experiments examined associations between hippocampal glutamate levels and striatal activity during reward processing. Therefore, discrepancies between these and our findings may be explained by differences in neuroimaging approach. For example, although reward-related ventral striatal activity has been related to dopamine release in this brain region (Knutson and Gibbs, 2007; Schott et al., 2008), it may not reflect presynaptic dopamine synthesis capacity.

We showed significant activity in the bilateral ventral striatum and significant connectivity between left ventral striatum and both lingual gyrus and hippocampus during reward anticipation. These results are in line with those of previous functional MRI studies, which have unequivocally implicated the ventral striatum as a key brain area involved in reward processing (Knutson et al, 2001; Bjork et al., 2004; Knutson and Cooper, 2005). In addition, several functional MRI studies examined functional connectivity of the ventral striatum in a reward context. For example, Schreiter and colleagues (2016) showed decreased functional connectivity between the left ventral striatum and anterior prefrontal cortex in patients with bipolar disorder during reward anticipation. Weiland et al. (2013) demonstrated increased connectivity between the ventral striatum and both paracentral lobule/precuneus and sensorimotor areas in youth with a family history of alcoholism during incentive anticipation (both reward and loss conditions). Unfortunately, whereas several studies reported group differences during reward anticipation, none of them specifically described striatal functional connectivity patterns in healthy individuals.

Because disturbed reward processing has been associated with both schizophrenia and addiction (Berridge, 2012; Berridge and Kringelbach, 2015; Radua et al., 2015; Luijten et al., 2017), our findings have potential implications for the understanding of these disorders. In particular, it is hypothesized that inappropriate attribution of incentive salience to otherwise relatively neutral environmental cues leads to the formation of psychotic symptoms (Heinz, 2002; Kapur, 2003). A leading contemporary preclinical model of psychosis proposes that these symptoms arise from a substantial decrease in the number of inhibitory parvalbumin-expressing GABAergic interneurons in the hippocampus, resulting in an overactive striatal dopamine system through manipulation of glutamatergic pyramidal neurons (Lodge and Grace, 2011; Grace, 2016). This is consistent with our findings that hippocampal glutamate levels were significantly correlated with reward anticipation-related ventral striatal activity, and inversely correlated with hippocampal coupling with ventral striatal function in the context of reward processing.

Some limitations have to be taken into account in interpreting the results of this study. First, we focused on reward anticipation and not on loss avoidance or feedback. This was because the anticipation phase has been most strongly linked with striatal dopamine function (Knutson and Gibbs, 2007; Schott et al., 2008). Moreover, striatal incentive findings with reward are generally more robust than those with loss avoidance (Knutson et al., 2001; Guyer et al., 2006). Second, the reported Glx signal is a composite peak, which not only incorporates glutamate but also its precursor glutamine. However, the Glx signal has been widely used as a marker of glutamatergic function, because it likely predominantly reflects glutamate levels, which are typically 5 to 6 times higher than those of glutamine (Kaiser et al., 2005). Third, MRS techniques cannot distinguish between intracellular and extracellular metabolite concentrations, and thus hippocampal metabolite levels as demonstrated in the current study reflect both.

In conclusion, our study shows for the first time that in healthy volunteers, higher hippocampal glutamate levels are correlated with reward anticipation-related ventral striatal activity, but inversely correlated with context-dependent functional connectivity between the anterior hippocampus and the ventral striatum during the anticipation of monetary reward. This suggests that human striatal responses to reward anticipation are influenced by hippocampal glutamate levels.
